# Radiotherapy and Its Intersections with Surgery in the Management of Localized Gynecological Malignancies: A Comprehensive Overview for Clinicians

**DOI:** 10.3390/jcm10010093

**Published:** 2020-12-29

**Authors:** Alexander Fabian, David Krug, Ibrahim Alkatout

**Affiliations:** 1 Department of Radiation Oncology, University Hospital of Schleswig-Holstein, Campus Kiel, Arnold-Heller-Str. 3, 24105 Kiel, Germany; david.krug@uksh.de; 2 Department of Obstetrics and Gynecology, University Hospital of Schleswig-Holstein, Campus Kiel, Arnold-Heller-Str. 3, 24105 Kiel, Germany

**Keywords:** minimally invasive surgical procedures, surgery, radiotherapy, ovarian neoplasms, endometrial neoplasms, uterine cervical neoplasms, vaginal neoplasms, vulvar neoplasms, survival analysis, quality of life

## Abstract

Surgery, including minimally invasive surgery, and radiotherapy are key modalities in the treatment of gynecological malignancies. The aim of this review is to offer the multidisciplinary care team a comprehensive summary of the intersections of surgery and radiotherapy in the local treatment of gynecological malignancies. Recent advances in radiotherapy are highlighted. Relevant publications were identified through a review of the published literature. Ovarian, endometrial, cervical, vaginal, and vulvar cancer were included in the search. Current guidelines are summarized. The role of radiotherapy in adjuvant as well as definitive treatment of these entities is synthesized and put into context with surgery, focusing on survival and quality of life. Although these outcomes have improved recently, further research must be focused on the number of life years lost, and the potential morbidity encountered by patients.

## 1. Introduction

**Figure 1:**
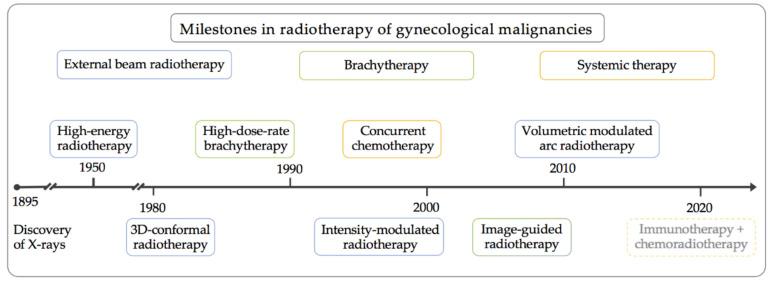
Milestones in radiotherapy for gynecological malignancies. A schematic timeline showing innovations in radiotherapy, including external beam radiotherapy, brachytherapy, and systemic therapy.

The last decades have witnessed significant advancements in surgical techniques for the treatment of gynecological cancers. The results are shorter hospital stays, less blood loss, and lower morbidity levels due to the minimally invasive approach [1,2,3,4]. Simultaneously, major innovations have transformed the field of radiation oncology (Figure 1) [5]. These innovations have led to more precise and more effective treatment by radiation therapy.

**Figure 2:**
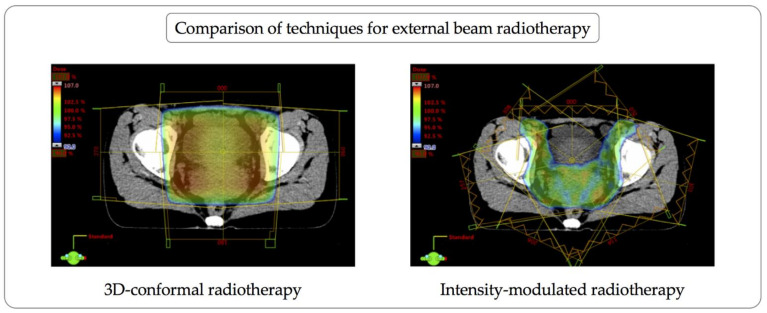
Comparison of techniques for external beam radiotherapy. FIGO stage IIIC cervical cancer in a 30-year-old woman treated with definitive radiotherapy including external beam radiotherapy, high-dose-rate brachytherapy, and concurrent cisplatin. She had laparoscopic nodal staging and ovarian transposition prior to radiotherapy. The actual intensity-modulated treatment plan (**right**), allowing for superior conformality, is juxtaposed to an alternative 3D-conformal plan (**left**). Inguinal nodes are included in the radiation field due to distal vaginal extension of the primary cancer.

The precision of treatment delivery was greatly enhanced by the introduction of three-dimensional conformal radiotherapy based on CT scans. This permitted computer-based delineation and the definition of target volumes as well as organs to be spared from external beam radiation (“organs at risk”). The next step in technical innovation was the introduction of intensity-modulated radiotherapy in the late 1990s, and volumetric-modulated arc radiotherapy by the 2000s. Both techniques employ multiple precise collimators (“multi-leaf collimator”) of linear accelerators to shape the radiation beam to the target volume and limit the dose to surrounding tissue. Using advanced treatment planning algorithms, the intensity of the dose is modulated to the treatment volume. This enhances the conformality of the prescription dose to the target volume. Organs at risk in close proximity to the target volume can be spared more effectively (Figure 2). Randomized controlled trials have shown that these techniques result in significantly lower toxicity levels and better health-related quality of life compared to older radiation therapy techniques for several anatomic sites. This is also true of gynecological cancers, as evidenced by lower gastrointestinal and urinary toxicity levels [6]. More precisely, grade 3 or higher acute gastrointestinal toxicity in cervical cancer patients was significantly reduced from 20–27.3% to 4.5–5% by intensity-modulated radiotherapy [7,8]. Similarly, grade 3 or higher acute genitourinary toxicity was significantly reduced from 15% to 5% [8]. Late toxicity is also significantly lower after intensity-modulated radiotherapy compared to older radiotherapy techniques [9]. However, as many as 11% of patients with cervical cancer may experience grade 3 or higher late gastrointestinal/urinary toxicity [9].

Modern radiotherapy techniques have been suspected to increase the incidence of radiation-induced secondary malignancies because of larger quantities of tissue receiving relatively low doses. Although this concern does not appear to hold true, the 15-year probability of a radiation-induced malignancy is estimated to be approximately 2% across treatment sites and entities [10,11]. In irradiated cervical cancer survivors, a large database study reported a standardized incidence ratio (observed vs. expected incidence) of 1.3 (95% CI, 1.28–1.33) for secondary malignancies [12]. This moderate risk of radiation-induced malignancies must be addressed when counseling patients before treatment.

In parallel with the advent of intensity-modulated radiotherapy and volumetric modulated arc radiotherapy, image-guided radiotherapy was introduced in the clinical setting [13]. Patients used to be positioned exclusively with skin marks, but modern linear accelerators have a built-in capacity to produce three-dimensional images with the treatment beam (megavoltage CT) or separate X-ray tubes and detectors (kilovoltage CT). Recently, two hybrid machines integrating a linear accelerator and magnetic resonance imaging were approved for clinical use [14]. While the results of magnetic resonance-guided external beam radiotherapy for gynecological malignancies are not available yet, the technique offers exciting prospects for daily adaptive radiotherapy and live imaging during treatment delivery without additional exposure to ionizing radiation.

Not only external beam radiotherapy but also brachytherapy has evolved and is now a field of active research. Brachytherapy is a key element in the treatment of gynecological cancers. Image-guided or magnetic resonance imaging-guided three-dimensional brachytherapy effectively reduces radiation doses to organs at risk and may also reduce toxicity [15,16,17].

The addition of chemotherapy enhanced the effectiveness in terms of overall survival and local control of radiotherapy-based approaches in multiple entities [18]. Typically, chemotherapeutic agents such as cisplatin are administered concomitantly during the course of radiotherapy. In fact, gynecological entities such as cervical cancer rank among those entities that benefit most from the addition of chemotherapy in terms of overall survival [18]. The purpose of combining radiotherapy and chemotherapy is to achieve “supra-additive” efficacy [19]. In other words, the combination is more effective in terms of tumor control than one would expect by the mere addition of each individual modality. Mechanistically speaking, tumor cells are more prone to radiation-induced DNA damage when exposed to chemotherapeutic agents that also interfere with DNA or its repair. Although normal tissue also experiences a higher level of acute toxicity, late toxicity is usually not, or just mildly increased after chemoradiotherapy compared to radiotherapy alone [20].

More recently, the combination of radiotherapy and immunotherapy has emerged as an attempt to further improve survival [21]. As radiotherapy harbors immunomodulatory effects that could enhance the effectiveness of immunotherapy, clinical trials have investigated their combination in multiple entities [22]. As shown in a phase-III trial, the immune checkpoint inhibitor durvalumab is associated with a significant survival benefit in patients with stage III non-small cell lung cancer who have undergone definitive chemoradiotherapy [23]. Several phase-I/II trials are currently investigating a similar approach in gynecological malignancies such as cervical cancer [24].

Taken together, innovations in the planning and delivery of radiotherapy as well as the delivery of concomitant chemotherapy have improved the therapeutic index of radiotherapy-based approaches by increasing efficacy and reducing toxicity. Radiotherapy continues therefore to be indicated as an adjuvant or alternative treatment to surgery in some gynecological cancers and clinical scenarios, whereas it may be obsolete in others. However, the various indications for radiotherapy and their relation to surgery may not be well known to all members of the multidisciplinary care team. Furthermore, a specialist in one field may find it difficult to consider all aspects that require attention in the interdisciplinary setting. Therefore, interdisciplinary tumor boards and the sharing of knowledge play an increasing role in the treatment of gynecological cancers. This comprehensive review summarizes the role of radiotherapy and its intersections with surgery for gynecological cancers. The review is intended to provide an overview for physicians outside the field of radiation oncology.

## 2. Methods

A narrative literature review was performed. This review focusses on newly diagnosed gynecological malignancies without distant metastases. The primary aim was to assess the current evidence and developments concerning radiotherapy and to put them into context with surgery in order to offer an overview for clinicians. The context of surgery was chosen, as both are local modalities that may complement each other. The most recent US-American National Comprehensive Cancer Network guidelines and European guidelines (ESMO, ESTRO, ESGO) were reviewed. In addition, PubMed/MEDLINE was searched for relevant studies in the English language with no time restriction (see Table S1). The search strategy included ovarian, endometrial, cervical, vaginal, and vulvar cancer. The intervention was radiotherapy, and outcomes were survival and quality of life. Studies were chosen by judgment of the authors and relevance to the multidisciplinary care team without preset eligibility criteria. Additional relevant studies were included from the personal reference databases of the authors. The search was conducted in July 2020.

## 3. The Role of Radiotherapy in Newly Diagnosed Localized Disease

### 3.1. Epithelial Ovarian Cancer

Ovarian cancer is the seventh most common type of cancer in women; the global 5-year survival rate for this entity is low at 30–40%. The highest incidence rates are 11.6/100,000 women in Central and Eastern Europe [25]. Most cases are of epithelial histology, and various subtypes exist [26]. Cytoreductive surgery and adjuvant systemic therapy are the mainstays of treatment for newly diagnosed cases, and have led to better survival rates [27]. Notwithstanding diverse opinions on the subject, the primary and most widely used surgical access is laparotomy. As shown in a meta-analysis of retrospective studies, minimally invasive surgery might yield similar survival outcomes compared to laparotomy. However, this thesis will have to be confirmed in prospective studies [28].

Neither US-American nor European guidelines recommend radiotherapy for epithelial ovarian cancer in the primary or adjuvant setting [29,30]. Historically, adjuvant whole abdominal radiotherapy has been used with the intention of reducing relapse rates and prolonging survival. Retrospective data suggest an absolute increase of 20% in 5-year disease-free survival for patients with FIGO stage IC and II by the addition of radiotherapy to surgery and chemotherapy [31]. However, the large majority of randomized controlled trials performed in the 1990s yielded no robust benefit from radiotherapy when compared to chemotherapy [32,33]. Although one randomized controlled study reported a significantly improved 5-year progression-free survival after adjuvant radiotherapy (56%) compared to chemotherapy (36%) in FIGO stage III patients, it also reported higher toxicity in the radiotherapy arm [34]. In these earlier trials, the authors employed radiotherapy techniques considered outdated in current times, because the procedures hardly permitted sparing of organs at risk. Recently, a prospective phase-II-trial reported favorable toxicity results for adjuvant whole abdominal radiotherapy using intensity-modulated radiotherapy in FIGO stage III ovarian cancer [35]. Yet, we still lack robust efficacy data for this approach. Therefore, adjuvant whole abdominal radiotherapy should not be used for the treatment of epithelial ovarian cancer.

### 3.2. Endometrial Cancer

Endometrial cancer of the uterine body is the fifth most common cancer in women and the most common gynecological cancer in high-income countries. Its incidence is highest in the USA at 19.1/100,000 women. Endometrial cancer usually occurs in postmenopausal women; obesity is a major risk factor [36]. As most patients are diagnosed in early stages by the presence of atypical vaginal bleeding, 5-year overall survival rates exceed 80% in high-income countries [37]. However, in patients with FIGO stage III or IV disease, the 5-year overall survival rate drops to approximately 57% or 20%, respectively [36]. Traditionally, endometrial cancer was classified as type I (endometrioid carcinoma) or type II disease (serous or clear-cell carcinoma) with distinct clinical and molecular features, although this may be regarded as a simplistic distinction [38]. Using advanced methods with genomic, transcriptomic, and proteomic characterization, molecular classification of endometrial cancer yielded four distinct molecular subtypes [39]. Recent data from the PORTEC-3 trial (see below) suggest that molecular subtypes may influence the choice of adjuvant therapy. This aspect will be addressed prospectively in the PORTEC 4a-trial for adjuvant vaginal brachytherapy [40,41,42].

Laparoscopic surgical staging is generally recommended, and includes hysterectomy with bilateral salpingo-oophorectomy with or without bilateral pelvic and para-aortic lymph node dissection [43,44]. The resulting tumor stage may be confined to the uterus (FIGO I), extend to the cervix (FIGO II), beyond the uterus (FIGO III), or invade adjacent organs with or without distant metastases (FIGO IV) [45]. Early-stage disease (FIGO stage I) is further subdivided into a low, intermediate, or high-risk subgroup based on the depth of invasion, histology, and grading [46]. Brachytherapy as well as external beam radiotherapy, each with or without chemotherapy, have been investigated extensively in different clinical scenarios of endometrial cancer.

In the adjuvant setting, vaginal brachytherapy is not indicated for FIGO stage I low-risk disease, but is usually recommended for intermediate-risk cases, especially in the presence of further risk factors (“high-intermediate risk”) [43,44]. This rationale was elaborated among others by the PORTEC-1 and PORTEC-2 trials. The PORTEC-1 trial studied adjuvant external beam radiotherapy versus observation in intermediate-risk cases [47]. No benefit was registered in overall survival, but intermediate-risk cases with additional risk factors had fewer local vaginal recurrences after external beam radiotherapy at the expense of higher toxicity. These results were confirmed by two other randomized controlled trials on external beam radiotherapy vs. observation [48,49]. Therefore, the PORTEC-2 trial studied external beam radiotherapy versus vaginal brachytherapy in high-intermediate risk patients. Although more pelvic recurrences were noted in the brachytherapy group, the vaginal control rate and overall survival rate were comparable [50]. However, brachytherapy was associated with significantly less toxicity and better health-related quality of life [51,52]. Concerning “real-world” data, vaginal brachytherapy was also associated with fewer recurrences as well as reduced mortality rates in a large US-American population-based analysis of FIGO stage I disease [53]. Brachytherapy was potentially underused compared to guideline recommendations.

Concerning high-risk patients, which include those with FIGO stage I (high risk subgroup) and FIGO II-IV disease, most guidelines recommend adjuvant external beam radiotherapy to the pelvis [43,44]. Important evidence has recently been added to the adjuvant management of these high-risk cases, as the role of systemic therapy with or without radiotherapy was unclear until then. The randomized phase-III-trial PORTEC-3 reported the results of adjuvant external beam radiotherapy with or without chemotherapy [54]. The study included FIGO stage I (high risk), stage II, and stage III cases. Overall 5-year survival rates were significantly improved with chemoradiotherapy (81.4% vs. 76.1%, *p* = 0.034), after two cycles of cisplatin during radiotherapy and four cycles of carboplatin/paclitaxel after radiotherapy compared to radiotherapy alone. Although grade 3 toxicity was similar, grade 2 toxicity was higher after chemoradiotherapy mainly due to peripheral neuropathy. This is reflected in health-related quality of life data at 12 and 24 months after treatment; most scales were similar between groups except for neurological symptoms [55].

The role of external beam radiotherapy was further refined by the randomized phase-III-trial GOG 258, in which adjuvant chemoradiotherapy was compared with adjuvant chemotherapy alone [56]. The large majority of patients included in the investigation had locally advanced FIGO stage III disease. The primary endpoint of the study, namely relapse-free survival, did not differ significantly between the two arms. Overall survival data were not available in the primary publication. At 5 years, vaginal recurrences (2% vs. 7%; HR 0.36) and pelvic or para-aortic nodal recurrences (11% vs. 20%; HR 0.43) were lower in the chemoradiotherapy group. However, distant recurrences (27% vs. 21%; HR 1.36) were more common after chemoradiotherapy compared to chemotherapy alone. Overall toxicity rates were comparable, but toxicity profiles differed. Health-related quality of life parameters were worse after chemoradiotherapy, but did not achieve the preset clinically meaningful difference. The essence of these data is that both modalities, radiotherapy and chemotherapy, are important for local and distant control, respectively. Their combination may prolong overall survival in high-risk patients. In fact, a registry-based study on FIGO stage III patients reported the increased use of chemoradiotherapy over time, which was accompanied by higher overall survival rates [57]. Further trials could elucidate whether a sequential or “sandwich” combination of radiotherapy and chemotherapy would reduce toxicity without compromising the prognosis [58]. Until then, shared decision-making remains the key factor in determining the appropriate adjuvant treatment modality for the individual patient.

The extent of external beam radiotherapy is strongly dependent on prior surgical evaluation of lymph nodes. Extension of the field of treatment to the para-aortic lymph nodes is associated with greater toxicity. The approach towards lymph node assessment varies considerably among institutions. There is a paucity of randomized trials comparing different approaches such as pelvic lymph node dissection with or without para-aortic lymph node dissection, sentinel lymph node biopsy, or no surgical nodal staging in different clinical scenarios. Each of these approaches may be associated with benefits, depending on the clinical scenario and the patient’s preference [59,60]. In accordance with recent clinical trials, para-aortic lymph nodes should be included in the radiation field only in patients with evidence of nodal spread to this site on imaging studies or pathological investigation [56].

Upfront primary radiotherapy is not recommended as a substitute for surgery because of the absence of appropriate evidence and the potentiality of a compromised prognosis. Radiotherapy, however, is used and recommended as primary treatment in patients who are medically unfit for surgery or when primary surgery is technically not feasible [43,44]. The treatment is usually based on brachytherapy with or without external beam radiotherapy, depending on the extent of the tumor. Although the majority of published reports in this regard are either retrospective or small prospective single-center studies, a systematic review of 2694 patients yielded promising results [61]. Grade 3 or poorer late toxicity rates were below 4%, and the disease-specific 5-year survival rate approached 80%, though highly dependent on the extent of disease. The rather favorable disease-specific survival is reflected in studies reporting as much as a 3.4-fold higher risk of dying due to intercurrent disease than due to endometrial cancer after primary radiotherapy [62,63].

The efficacy of a primary radiotherapy-based approach may be enhanced by the addition of chemotherapy, although the patient’s tolerance of an intensified treatment regimen could be limited in this medically unfit population. Larger prospective multicenter trials will be needed to validate the efficacy and elucidate the impact of this approach on health-related quality of life in patients who receive primary radiotherapy for endometrial cancer.

To sum up, adjuvant brachytherapy should be considered in intermediate risk FIGO stage I disease with additional risk factors to reduce vaginal recurrences (Table 1). External beam radiotherapy combined with chemotherapy is indicated in many high-risk patients in order to improve local control and overall survival. Primary radiotherapy is an option in patients who are not eligible for surgery (Table 2).

### 3.3. Cervical Cancer

Cervical cancer is the fourth most common cancer in women. While the global incidence is 13.1/100,000 women, low- and middle-income countries have markedly higher rates of 75/100,000 women [71]. As the incidence has already dropped in countries with effective screening programs, a further decrease is expected with the implementation of HPV vaccination [72,73]. The most common histological subtype is squamous cell carcinoma. Adenocarcinoma may be associated with a poorer prognosis [74]. The FIGO staging system was revised in 2018 [75]. The tumor may be confined to the uterine cervix (FIGO I), extend beyond the uterus (FIGO II), extend further into the true pelvis and/or with lymph node involvement (FIGO III), or invade the bladder or rectum with or without distant metastases (FIGO IV). Depending on the presence of risk factors, each of these stages is divided into further subgroups.

Generally, most patients with FIGO stage I disease are treated with surgery [76,77,78]. Surgical approaches include conization, trachelectomy, and (radical) hysterectomy with or without lymph node dissection. The surgical approach in the individual patient depends on risk factors and the resulting substage, the patient’s desire to preserve her fertility, and local expertise. Guidelines suggested minimally invasive surgery or laparotomy for radical hysterectomy in early stages [78]. Minimally invasive surgery was routinely used before the availability of randomized data compared to open surgery [79]. Surprisingly, however, minimally invasive surgery is associated with significantly poorer survival outcomes, as reported recently in a randomized trial and a large cohort study [80,81]. The former trial randomized 613 women with cervical cancer of FIGO IA1 (and lymphovascular invasion) to FIGO IB1 either to minimally invasive surgery or open surgery. At a median follow up of 2.5 years, the 3-year overall survival was worse after minimally invasive surgery compared to open surgery (93.8% vs. 99%; HR 6.00; CI, 1.77–20.30). Of 34 recurrences, all women except one had stage IB1 disease of grade 2 or higher. Therefore, the role of minimally invasive surgery for early stages may be debated. However, only about 8% of the patients in both groups had early stages of IA1 or IA2. In addition, the survival benefit after open surgery was still present even after adjusting for stage of disease [80]. Therefore, the minimally invasive access was more or less abandoned in the surgical treatment of cervical cancer. One of its disadvantages is that, despite its widespread popularity, many surgeons may lack the experience to perform these challenging and financially attractive operations. Recently, retrospective data on the influence of surgeon volume and experience was reported. A study that focused on robot-assisted minimally invasive surgery reported less local recurrences for tumors smaller than 2 cm in the absence of adjuvant chemoradiotherapy in more experienced centers [82]. Cusimano and colleagues, however, still observed inferior survival after minimally invasive surgery for stage IB1 cases compared to open surgery, even after adjusting for surgeon volume [83]. Despite the large number of suggested explanations, we do hence not know the exact reasons for different survival outcomes after open or minimally invasive surgery in cervical cancer patients [84]. Until the problem is resolved, we will have to accept the retrograde step in oncological surgery and may even be compelled to review the surgical access for endometrial cancer.

The higher the substage of the cervical cancer, the more primary radiotherapy is an alternative for example in stage IB2 or even preferred to surgery as in stage IB3 (Table 2) [76]. The combination of surgery and radiotherapy causes excessive morbidity [85]. As higher stages may require adjuvant radiotherapy after initial surgery, and as primary radiotherapy results in at least equivalent survival rates, primary radiotherapy is usually preferred from stage II on [76,85,86,87,88]. Primary radiotherapy includes external beam radiotherapy, brachytherapy, and concomitant chemotherapy with cisplatin.

Whether a primary surgical or a primary radiotherapy-based approach should be given preference for stage IB2-IIB disease is a matter of active research and discussion. Neoadjuvant chemotherapy prior to surgery was superior to radiotherapy alone [89]. Radiotherapy alone, however, is outdated in most cases because the addition of concomitant chemotherapy significantly improves overall survival [67]. Two randomized phase-III-trials recently reported outcomes of neoadjuvant chemotherapy plus surgery versus chemoradiotherapy in stage IB2-IIB disease [68,90]. The multicenter European study showed similar overall survival outcomes, but the surgical arm had significantly more acute toxicity levels of grade 3 or higher (35% vs. 21%; *p* < 0.001). In addition, 36% of patients in the surgical arm needed adjuvant radiotherapy, whereas only 3 % had additional surgery in the radiotherapy arm [68]. The single-center Indian trial also reported similar overall survival rates. The 5-year disease-free survival, however, was significantly better in the radiotherapy arm (69.3% vs. 76.7%; *p* = 0.038). At 24 months after treatment, there was no difference in toxicity except for vaginal toxicity, which was higher after radiotherapy (12% vs. 26%; *p* > 0.001) [90]. Viewed together, the results of these studies appear to favor a primary radiotherapy-based approach in stage IB2-IIB disease. Furthermore, it would be reasonable to state that current toxicity rates are lower than those observed in these trials, because intensity-modulated external beam radiotherapy and image-guided brachytherapy were either omitted altogether or not used on a routine basis [9].

The question as to whether neoadjuvant chemotherapy before primary chemoradiotherapy could improve outcomes was tested in a recent phase-III-trial. Surprisingly, overall survival was significantly poorer in the group that received neoadjuvant chemotherapy compared to chemoradiotherapy alone [91]. Therefore, concurrent chemoradiotherapy remains the standard of care for locally advanced disease.

Although the combination of surgery and radiotherapy is generally avoided in the treatment setting as mentioned above, it may be used for staging in the management of para-aortic lymph nodes. When clinical staging reveals the involvement of pelvic lymph nodes, the risk of para-aortic lymph node dissemination is high and is associated with a poorer prognosis [92,93]. Extension of the field of radiotherapy to the para-aortic lymph nodes up to the level of the renal vessels increases toxicity, albeit less so with intensity modulated radiotherapy [94]. Hence, if pelvic lymph nodes are affected but para-aortic spread is unclear or even negative per clinical staging, laparoscopic para-aortic lymph node staging may be performed prior to primary chemoradiotherapy, as stated in current guidelines [76,77,95]. The field of radiotherapy is usually only extended in cases of evident para-aortic lymph node disease; prophylactic extension of the field remains controversial [96,97,98]. This approach is supported by a study reporting favorable survival rates in locally advanced cases with a negative PET-CT for para-aortic involvement; the patients underwent laparoscopic staging and the field of radiotherapy was defined subsequently [99]. The laparoscopic approach was either extra- or transperitoneal and included lymphadenectomy from the aortic bifurcation to the left renal vein. The field of radiotherapy was extended to the para-aortic region if para-aortic involvement of any size was present per histology. This was the case in 12% (29/237) of the patients. Routine clinical staging versus laparoscopic lymph node dissection for FIGO (2009) IIB-IVA cases was investigated in just one randomized study [100]. In the arm of clinical staging, suspicious para-aortic lymph nodes were biopsied via CT-guidance. Biopsies revealed para-aortic lymph node metastases in 8% of the cases (9/114). In the arm of surgical staging, the surgical approach was mostly laparoscopic and extra- or transperitoneal. Of note, the field of lymphadenectomy encompassed pelvic lymph nodes as well as para-aortic lymph nodes up to the renal vessels. Lymphadenectomy resulted in an upstaging in 33% (39/120) and in the detection of positive para-aortic lymph nodes in 24% (29/120) of the cases. In histologically proven para-aortic involvement of any size, the field of radiation was extended to the para-aortic region in both arms. Patients did not undergo routine PET-CT imaging in this study. Although the acute toxicity profile was low after surgical staging, there was no statistically significant difference in overall survival per randomized arm [100,101]. Similarly, a recent retrospective cohort analysis suggested no survival benefit after surgical staging compared to clinical staging [102]. Depending on its availability, PET-CT is now the standard of care for staging in some countries. Therefore, we need randomized studies comparing surgical staging versus clinical PET-CT-based staging. Until then, surgical staging remains an option for individualized treatment, especially if PET-CT imaging is not routinely available.

Local dose escalation to the cervix is an essential component of the primary radiotherapy-based approach. This is usually accomplished by intracavitary brachytherapy. Paralleling the premature adoption of minimally invasive surgery and in the absence of randomized data, advanced external beam radiotherapy techniques were used to an increasing extent as a substitute for brachytherapy [103]. Stereotactic external beam radiotherapy delivers high ablative doses to the tumor at steep dose gradients to surrounding normal tissue. Diverse data have been reported on dose escalation by stereotactic radiotherapy. While one population-based study reported equivalent survival rates, another showed poorer survival compared to brachytherapy [103,104]. In addition, a single-arm phase-II-trial of stereotactic radiotherapy for dose escalation was terminated early due to excessive toxicity [105]. Particle radiotherapy, including protons or carbon ions, is a further possible technology for radiation dose escalation and its outcomes continue to be investigated [106,107].

Another strategy for the local intensification of treatment was tested in a phase-III-trial in which brachytherapy was compared with radical hysterectomy after external beam radiotherapy. Additional surgery yielded no benefit in terms of local control or survival [108]. Taken together, brachytherapy remains the standard of care for dose escalation; its outcomes continue to improve with the implementation of image guidance for planning treatment [109].

In the postoperative adjuvant setting of early-stage cervical cancer, guidelines recommend observation alone or adjuvant radiotherapy with or without chemotherapy in the presence of risk factors (Table 1) [76,77]. Adjuvant external beam radiotherapy in FIGO IB2 patients with two additional risk factors (e.g., tumor size > 4 cm, deep stromal invasion) is associated with significantly fewer local relapses and significantly better progression-free survival, as shown in a randomized phase-III-trial [64]. However, when pathological staging after radical hysterectomy yields nodal involvement, positive resection margins or parametrial invasion, adjuvant chemoradiotherapy should be given preference because the risk of recurrent disease is high in these patients [76,77]. Moderate evidence suggests a survival benefit from the addition of concomitant chemotherapy to external beam radiotherapy in the adjuvant setting as well [110]. Additional vaginal brachytherapy is an option in patients with a high risk of vaginal recurrence [77]. Indeed, brachytherapy is associated with a significantly better overall survival, especially in the presence of positive resection margins [111]. However, when the initial work-up shows that the patient would probably need adjuvant radiotherapy, the guidelines recommend a primary radiotherapy-based approach rather than primary surgery [77].

In conclusion, primary surgery is the treatment of choice for early stages of cervical cancer, whereas primary chemoradiotherapy is recommended at least from FIGO stage II on. Brachytherapy is essential in a primary radiotherapy-based approach. Although the role of laparoscopic para-aortic nodal staging is unclear, it may be used to define the extent of the field of radiation.

### 3.4. Vaginal Cancer

With an incidence of 1/100,000 women, primary vaginal cancer is a rare entity [112,113]. Most cases are squamous cell carcinomas and 65% are associated with human papillomavirus infection [114]. According to the FIGO stages revised in 2009, the tumor may be confined to the vagina (FIGO I), invade the pelvis to a limited degree (FIGO II), invade the pelvis to a greater extent such as reach the pelvic wall and/or spread to lymph nodes (FIGO III), or invade adjacent organs and/or spread to distant organs (FIGO IV) [115]. Due to the rarity of primary vaginal cancer and the consequent lack of randomized data, the treatment approach is highly individualized and similar to that for cervical cancer. We have a small number of guidelines on the subject, mainly based on “expert consensus” [116,117].

Stage I vaginal cancer can be treated surgically by wide excision [118]. A radiotherapy-based approach is an alternative. In fact, from stage II on most cases are treated with primary radiotherapy in order to achieve organ preservation [115]. A primary radiotherapy-based approach usually includes external beam radiotherapy, brachytherapy, and concurrent chemotherapy as in cervical cancer. The radiation field encompasses the pelvic lymph nodes and should be extended to the inguinal lymph nodes if the primary is located in the lower third of the vagina [115]. As in cervical cancer, brachytherapy is also an integral part of the primary radiotherapy-based approach for vaginal cancer. According to US-American guidelines, it should be used if the tumor exceeds 0.5 cm in thickness [117]. A large registry-based study confirmed the overall survival benefit of adding brachytherapy to external beam radiotherapy, regardless of tumor stage [119]. Furthermore, image-guidance is increasingly used for brachytherapy in vaginal cancer because it may be associated with better local control [120,121]. Retrospective data revealed that the concurrent administration of chemotherapy is associated with a survival benefit [122]. Therefore, cisplatin should be considered in a primary radiotherapy-based approach.

Data on toxicity and quality of life are scarce because of the retrospective nature of most studies. However, one retrospective single-center study reported overall grade 3 or 4 toxicity rates of 23% after primary radiotherapy [123]. This must be taken into account when planning treatment for the individual patient. Analogous to cervical cancer, minimally invasive surgical nodal staging may be performed prior to primary radiotherapy [115]. Minimally invasive surgery may also be offered for ovarian transposition in order to prevent radiation-induced dysfunction [115].

Taken together, the treatment approach for primary vaginal cancer is similar to that for cervical cancer because of the paucity of prospective data for this rare entity. Early stages can be treated by surgery. Primary chemoradiotherapy should be offered as an alternative or in more advanced stages in order to achieve organ preservation.

### 3.5. Vulvar Cancer

Vulvar cancer is uncommon and affects less than 2/100,000 women [124]. Two subgroups have been described. Vulvar cancer at a younger age is associated with human papillomavirus infection, whereas this association is less frequent in older women [125]. The 2000’s witnessed an increase in the incidence of the disease in younger patients [124]. Most cases are of squamous cell histology [125]. The most prominent prognostic factor is nodal status because the 5-year overall survival is 84% in node-negative cases, compared to 30% in cases with three or more positive lymph nodes [126].

Early stages are usually treated surgically with superficial or radical (partial) vulvectomy, depending on tumor size and location, as stated in the guidelines [127,128]. In cases of positive resection margins of the primary tumor, re-excision is recommended if feasible. If this is not feasible due to imminent exenteration or if the resection margins remain positive, adjuvant radiotherapy to the primary tumor is recommended [127]. A large registry-based analysis supports this approach: a significant 3-year overall survival benefit was registered after adjuvant radiotherapy to the primary site compared to no radiotherapy (67.4% vs. 58.5%, *p* < 0.001) [65].

Due to its impact on prognosis, the assessment and management of regional lymph nodes is also important in early-stage disease with a clinically negative nodal status. Nodes should be assessed by surgery; either an inguinofemoral lymphadenectomy or a sentinel lymph node biopsy should be performed [127,128]. The latter should be restricted to smaller (<4 cm) and unifocal primary tumors without clinical evidence of nodal spread [128]. Surgical lymph node evaluation may be ipsilateral or bilateral, depending on the size and location of the primary tumor. The optimal adjuvant treatment approach concerning radiotherapy in cases of positive lymph nodes is a debated issue because the data on the subject is largely retrospective in nature (Table 1) [129].

When the sentinel lymph node biopsy reveals a singular micrometastasis (<2 mm), a US-American guideline recommends radiotherapy as an alternative to inguinofemoral lymphadenectomy [127]. Conversely, a less recent European guideline solely suggests inguinofemoral lymphadenectomy in this scenario [128]. However, a multicenter phase-II trial recently reported only two inguinal recurrences in 129 patients treated with radiotherapy alone after the detection of a micrometastasis by sentinel lymph node biopsy [69]. Given the favorable toxicity profile of only 4.2% grade 3 toxicity, radiotherapy appears to be an appropriate alternative in the presence of a singular micrometastasis after sentinel lymph node biopsy, although randomized data on the subject are lacking. When a singular metastasis larger than 2 mm is detected per sentinel lymph node biopsy, guidelines recommend complete inguinofemoral lymphadenectomy [127,128].

In cases of two or more positive lymph nodes per lymphadenectomy or extracapsular extension, US-American and European guidelines suggest adjuvant radiotherapy to inguinal and pelvic lymph nodes in order to reduce local recurrences and improve survival [127,128]. The role of adjuvant radiotherapy was confirmed in a prospective trial in which patients with inguinal lymph node involvement were randomized either to adjuvant radiotherapy encompassing the groin and pelvis, or to pelvic lymphadenectomy [66]. The radiotherapy group had a significant survival benefit in cases of upfront clinically suspected and/or more than two affected lymph nodes. Similarly, a large multicenter cohort study showed an overall survival benefit after adjuvant radiotherapy when two or more nodes were positive [130]. Concurrent chemotherapy may be added when the risk of recurrence is rated very high due to bulky disease, extracapsular extension, or residual tumor [127]. Conversely, retrospective data revealed that adjuvant radiotherapy was not associated with better overall survival in cases of a single lymph node metastasis without extracapsular extension [130,131]. Therefore, adjuvant radiotherapy is usually not recommended in the latter scenario, although used quite often [127,128,130].

In locally advanced cases, neoadjuvant or definitive chemoradiotherapy are appropriate alternatives when complete resection is not feasible (Table 2) [127]. The field of radiotherapy should include the primary tumor as well as pelvic lymph nodes. If surgical lymph node staging was performed in the groin and yielded no evidence of nodal spread, this site may be omitted from the radiotherapy field; otherwise the treating physician should include inguinal lymph nodes in the field of radiotherapy [127]. Cisplatin is given preference in concurrent chemotherapy [127]. Prospective and retrospective data support the use of primary chemoradiotherapy in this setting. A prospective phase-II trial assessed the rate of complete clinical and pathological response after primary chemoradiotherapy in unresectable T3/T4 cases [70]. Sixty-four percent (37/58) of patients achieved a complete clinical response and, of those who had a confirmatory biopsy, 78% (29/34) experienced a complete pathological response. A retrospective study of 26 women treated with intensity-modulated radiotherapy and concomitant cisplatin reported a high complete response rate of 80.7% [132]. Complete response after chemoradiotherapy is associated with fewer recurrences and longer overall survival [132,133]. A large registry-based study of locally advanced cases reported similar survival rates in women treated with primary chemoradiotherapy compared to neoadjuvant chemoradiotherapy followed by surgery [134]. Although primary chemoradiotherapy appears to be effective, the associated toxicity must be taken into account. In the afore-mentioned phase-II-study, 15.5% (9/58) of the patients discontinued the treatment early due to toxicity [70]. In a retrospective study, 19% of patients had grade 3 or 4 toxicity after primary chemoradiotherapy [132]. We lack patient-reported quality of life data in this setting.

In vulvar cancer, radiotherapy is an alternative to inguinofemoral lymphadenectomy in the presence of a singular lymph node micrometastasis. Furthermore, adjuvant radiotherapy improves overall survival in cases of positive resection margins of the primary or extensive nodal spread. Neoadjuvant chemoradiotherapy followed by surgery or definitive chemoradiotherapy are effective in advanced disease.

## 4. Conclusions

Women with newly diagnosed gynecological malignancies require a multidisciplinary care team to ensure optimal treatment. This comprehensive review aimed to explore the manifold intersections between surgery, including minimally invasive surgery, and radiotherapy in the light of recent advances and challenges. As radiotherapy and surgery are both local treatments, they do complement one another in some clinical scenarios, but also compete in others.

Adjuvant radiotherapy after surgery is generally indicated for many gynecological malignancies in the presence of risk factors for adverse oncological outcomes. Adjuvant radiotherapy reduces local relapses and ideally improves overall survival. Primary radiotherapy instead of surgery is a well-accepted and extensively investigated alternative for many patients with cervical or vaginal cancer. Minimally invasive surgery can complement this treatment approach for example by protective ovarian transposition or by determining the field size of radiotherapy after para-aortic nodal staging. Concerning endometrial and vulvar cancer, primary radiotherapy is the preferred option in localized but unresectable disease. Furthermore, modern radiotherapy techniques have reduced treatment-related toxicity, whereas concomitant chemotherapy improves overall survival in many scenarios. Any attempt to further improve the therapeutic ratio of a modality should be approached with caution, as proven by the premature adoption of minimally invasive surgery or stereotactic radiotherapy for cervical cancer prior to the attainment of high-quality data on the subject [79,80,103]. Our review highlighted the absence of patient-reported quality of life data, especially in the treatment of rare entities that require further comparative study. The latter holds true for surgery, including minimally invasive surgery, as well as radiotherapy.

To conclude, advances in surgery as well as radiotherapy contribute to improved outcomes in the treatment of gynecological malignancies if implemented carefully. The number of life years lost, however, is still significant and justifies ongoing efforts from all members of the scientific and clinical team in the field of gynecologic oncology.

## Figures and Tables

**Table 1:** Schematic overview of common indications for adjuvant radiotherapy in newly diagnosed localized gynecological malignancies. Adjuvant radiotherapy is not used for ovarian cancer. The approach to vaginal cancer is similar to that for cervical cancer. Ovarian and vaginal cancer are therefore not listed here.

Entity	Endometrial Cancer		Cervical Cancer	Vulvar Cancer	
**Postoperative** **clinical scenario**	High-interm. risk in early stage	High risk in early stage or advanced stage	Risk factors (e.g., tumor > 4 cm, deep stromal invasion, positive resection margins)	Primary tumor with persistent positive resection margins	Lymphadenectomy with ≥2 positive LN or ECE
**Radiotherapy**	BT	EBRT +/− Chemo	EBRT +/− Chemo +/− BT	EBRT	EBRT
**Most relevant available efficacy outcome**	Vaginal recurrence	Pelvic/Vaginal recurrence, overall survival	Local recurrence, progression-free survival	Overall survival	Overall survival
**Corresponding** **Publications**	Prospective, randomized [46,49]	Prospective, randomized [53,55]	Prospective, randomized [64]	Retrospective [65]	Prospective,randomized [66]

Abbreviations: +/−, with or without; BT, brachytherapy; EBRT, external beam radiotherapy; LN, lymph node; ECE, extracapsular extension.

**Table 2:** Schematic overview of common indications for primary radiotherapy in newly diagnosed localized gynecological malignancies. Primary radiotherapy is generally not used for ovarian cancer. The approach towards vaginal cancer is similar to that for cervical cancer. Ovarian and vaginal cancer are therefore not listed here.

Entity	Endometrial Cancer	Cervical Cancer	Vulvar Cancer	
**Clinical scenario**	Medically or surgically inoperable	FIGO stage > IB2 or >II	Singular micrometastasis (≤2 mm) in SLN-biopsy	Medically or surgically inoperable
**Radiotherapy**	BT +/− EBRT +/− Chemo	EBRT + BT + Chemo	EBRT to the groin	EBRT + Chemo
**Most relevant available** **efficacy outcome**	Disease-specific survival	Overall survival	Local recurrence	Complete response rate
**Corresponding** **Publications**	Retrospective [60]	Prospective, randomized [67,68]	Prospective, non-randomized [69]	Prospective, non-randomized [70]

Abbreviations: +/−, with or without; BT, brachytherapy; EBRT, external beam radiotherapy; SLN, sentinel lymph node.

## Data Availability

No new data were created or analyzed in this study. Data sharing is not applicable to this article.

## Supplementary Materials

The following are available online at https://www.mdpi.com/2077-0383/10/1/93/s1. Table S1: Search strategy—PubMed/MEDLINE.
