# Supplementary material for: Radiotherapy and Its Intersections with Surgery in the Management of Localized Gynecological Malignancies: A Comprehensive Overview for Clinicians
Source: J Clin Med. 2020 Dec 29;10(1):93. doi: 10.3390/jcm10010093 (PMC7796321; doi:10.3390/jcm10010093)
Supplement: Supplementary file 1 [file jcm-10-00093-s001.pdf]

Table S1. Search strategy - PubMed/MEDLINE.

Population		Intervention		Comparator		Outcome	Hits
"Ovarian Neoplasms"[Mesh]		"Radiotherapy"[Mesh]		n.a.		("Survival Analysis"[Mesh] OR "Quality of Life"[Mesh])	206
"Endometrial Neoplasms"[Mesh]		"Radiotherapy"[Mesh]		n.a.		("Survival Analysis"[Mesh] OR "Quality of Life"[Mesh])	398
"Uterine Cervical Neoplasms"[Mesh]	AND	"Radiotherapy"[Mesh]	AND	n.a.	AND	("Survival Analysis"[Mesh] OR "Quality of Life"[Mesh])	962
"Vaginal Neoplasms"[Mesh]		"Radiotherapy"[Mesh]		n.a.		("Survival Analysis"[Mesh] OR "Quality of Life"[Mesh])	60
"Vulvar Neoplasms"[Mesh]		"Radiotherapy"[Mesh]		n.a.		("Survival Analysis"[Mesh] OR "Quality of Life"[Mesh])	62

The search was conducted in July 2020.
